# A 27-Year Experience With Day Surgery Transurethral Resection of the Prostate

**DOI:** 10.7759/cureus.55699

**Published:** 2024-03-07

**Authors:** Tasciana T Gordon, Neil Gordon

**Affiliations:** 1 General Surgery, Mater Hospital, Brisbane, AUS; 2 Urology, Cairns Private Hospital, Cairns, AUS

**Keywords:** day surgery transurethral resection of the prostate, day surgery, catheterisation, prostatectomy, bladder outlet obstruction, transurethral resection of prostate, turp

## Abstract

Introduction

Transurethral resection of the prostate (TURP) is the standard surgical procedure for obstructive symptoms of the lower urinary tract when medical management fails. Progression in TURP procedures has led to reduced catheterization time following transurethral prostatic resection. This study describes the methods and results of TURP performed in the day surgery setting.

Materials and methods

This retrospective study was performed at a day surgical hospital serving a patient population of more than 200,000 people. Over a 27-year period, a total of 1,123 patients with a mean age of 73.6 years (range: 49 to 91 years) underwent same-day conventional (electrosurgical monopolar) transurethral prostatic resection. Of the procedure, 43 patients (11%) received spinal anaesthesia, and the remainder received general anaesthesia.

Results

Over the years, there has been an increase in the use of medication to manage bladder outflow obstructive symptoms, which has led to the preoperative post-micturition volumes of urine being increased (>200 ml) at the time of surgical intervention. The mean American Urological Association (AUA) score was 22 (range: 10-35). Due to the reduced bladder tone preoperatively and the noted intraoperative distension of the bladder, early catheter removal is contraindicated in these patients. The mean duration of catheterization was 6.4 days (range: two to 28 days). No patient was readmitted to the hospital for retention of urine. However, 11 patients in the series had re-catheterization due to failure of micturition after the removal of the catheter. No patients were admitted to the hospital for clot retention or sepsis postoperatively. This resulted in the patients being discharged home with a catheter in place, which became our standard practice.

Conclusion

Conventional transurethral resection of the prostate can be effectively managed in the day surgery setting with minimal morbidity. This improves the patient’s quality of life as well as the burden on hospital costs. Additionally, the outpatient nature of day surgery may lead to decreased overall healthcare expenses for both the patient and the healthcare system. As healthcare systems continue to prioritize streamlined and patient-centred approaches, day surgery for TURP emerges as a viable and advantageous option.

## Introduction

Bladder outlet obstruction surgery constitutes approximately 25% of a urologist's workload and stands as the predominant major pathology within the field. Despite various proposed methods for treating bladder outflow obstruction, transurethral resection of the prostate (TURP) remains the established benchmark for comparison [1]. After Guyon's inaugural transurethral prostatic resection at Necker Hospital in Paris in 1901, this technique surpassed open prostatectomy, emerging as the favoured option for more than 95% of patients [2]. The evolution of modern electrosurgical transurethral prostatic resection can be traced back to Stern's description in 1926, later refined by McCarthy in 1931 [3].

Surgical treatment of bladder outlet obstruction has seen progress with the introduction of various methods, including transurethral needle ablation, visual laser ablation (Greenlight®), transurethral vaporization, transrectal high-intensity focused ultrasound, transurethral microwave thermotherapy, and Urolift® [4]. Innovative treatments for bladder outlet obstruction aim to minimize both morbidity and the duration of hospitalization linked to TURP. As noted by Cherrie et al., a drawback of TURP in contrast to several contemporary therapies for benign prostatic hypertrophy (BPH) is the requirement for postoperative catheterization. This necessity, in turn, impedes the potential for an early discharge home following the procedure [5]. Several studies have documented the prompt removal of catheters after transurethral prostatic resection. However, although early catheter removal is desirable, it can result in the need for re-insertion if micturition is not successful [4-7]. Previous experience with planned in-patient TURP has led to the conclusion that preoperative post-micturition volumes greater than 200 mL are likely to result in incomplete emptying of the bladder as a result of reduced bladder tone [4]. This is also contributed to by prolonged bladder distention during the continuous flow technique of the procedure.

Appropriate home-care instructions, explanation, and demonstration of the catheter by the surgeon, reinforced by the preoperative nurse, combined with written information explained and provided to the patient by the postoperative nurse, resulted in minimal incidents. In addition, 24-hour telephone access to the surgeon was made available.

The decision regarding catheter removal is contingent upon parameters encompassing the patient's cognitive acuity, adequacy of fluid intake, regular bowel movements, and characteristics of catheter drainage [4]. Advances in contemporary anaesthesia facilitate the expedited satisfaction of these criteria in the immediate postoperative period [4]. Transurethral resection of the prostate as a day procedure stands as a clinically viable and efficacious alternative.

## Materials and methods

This retrospective study was performed over a 27-year period (1997-2024, inclusive) at Cairns Day Surgery, Cairns, Australia, a day surgical hospital serving a patient population of more than 200,000 people. The facility had the capacity for extended observation. For patients requiring inpatient management or intensive care, the adjacent parent private hospital facility was accessible.

Inclusion criteria involved patients undergoing transurethral prostatic resection by a single surgeon at the day surgical hospital, patients with increased preoperative post-micturition volumes of urine (post-void residual volume >100 mL), an American Urological Association (AUA) score >15, and failure of medical therapy. patients who did not undergo conventional transurethral prostatic resection, cases with incomplete or missing medical records, patients with a history of other major urological procedures, and those with contraindications to spinal or general anaesthesia were excluded from the study.

In the day surgical unit, 1,123 consecutive patients with significant bladder outlet obstructive symptoms or urine retention underwent elective TURP. The postoperative assessment included AUA symptom scores (Appendix 1). Among these patients, 37 individuals (3.29%), with a mean age of 75.5 years (range: 49 to 81 years), underwent surgery due to urinary retention following prior catheterization at the public hospital emergency department. The decision for elective surgery was based on symptom severity, AUA score, urodynamic studies utilizing the Laborie System® (Laborie, Portsmouth, NH), failed medication (alpha-adrenoreceptor antagonist medications, 5-alpha-reductase inhibitors, or combined medications), and a history of obstructive symptoms leading to urine retention.

The preoperative evaluation, including digital rectal examination, full blood examination, serum urea, electrolytes, creatinine, prostate-specific antigen, urinary tract ultrasound, and urinalysis, was completed. Only patients with abnormal urinalysis were sent for microbiological studies. All patients were administered their usual medications for other medical conditions. In no case was aspirin or other anti-inflammatory medication stopped preoperatively. Patients taking anticoagulants such as rivaroxaban, apixaban, or dabigatran were asked to stop taking them four days preoperatively. Warfarin was ceased three days preoperatively and resumed when the catheter was removed. No patients suffered any adverse results because of the anti-coagulation cessation; haematologists were consulted for patients with a high risk of thrombosis.

The patients were admitted on the morning of the procedure after fasting for four to six hours. They were instructed to drink 300 to 400 mL of water four hours preoperatively. Forty-three procedures were performed with the patient under spinal anaesthesia (11%) initially due to the preference of the anaesthetist, and the remainder under general anaesthesia. It was noted that there was an increased recovery with spinal anaesthesia, so practice was adapted to only use general anaesthesia for the remainder of the patients. A single urologist performed all resections. The anaesthetic technique was according to the individual anaesthetist’s protocol. 

The procedure was carried out under antibiotic prophylaxis with gentamicin 3 mg/kg stat and postoperatively either with trimethoprim, norfloxacin, ciprofloxacin, amoxicillin/clavulanic acid, or sulfamethoxazole/trimethoprim oral medication, depending upon the sensitivity of preoperative positive urine cultures and the patient’s allergy list.

A STORZ resectoscope (27-Ch continuous flow, KARL STORZ, Tuttlingen, Germany) with a visual obturator and active working element was utilized for all resections [4]. The irrigation during the procedure consisted of 1.5% weight per volume of glycine. The electrosurgical unit employed was a CONMED Aspen Excalibur Plus (CONMED Corporation, Largo, FL), with a standard STORZ loop electrode set at 180W pure cutting current and 80W coagulation current [4]. Coagulation did not involve the use of roller-ball electrodes.

Transurethral resection of the prostate followed the standard technique [4]. At the procedure's conclusion, a 22-Ch all-silicone three-way catheter was inserted, with most patients receiving 30 mL of water in the catheter balloon. For patients with bleeding from venous sinuses, catheter traction was applied, and 60 ml of water was instilled in the balloon. Catheter traction was consistently applied for one hour postoperatively in the majority of cases to ensure hemostasis.

Postoperatively, continuous irrigation of the catheters was done with a 0.9% saline solution. The decision to cease irrigation was made by a registered nurse, considering factors such as patient mental alertness, oral fluid intake adequacy, and the colour of catheter drainage. Each patient underwent an assessment by the urologist four to eight hours postoperatively before discharge.

## Results

There were a total of 1,123 patients included in the study, with an average age of 73.6 years (range: 49 to 91 years). The mean AUA symptom score was 22 (range: 10 to 35). The mean resection weight was 22.48 grams (range: 7-81 grams). Among them, 43 patients received spinal anaesthesia, while the remaining 1,080 underwent general anaesthesia. Routine catheter traction was applied for one hour. In patients with same-day catheter removal, the duration of catheterization was 7.69 hours for spinal anaesthesia and 3.86 hours for general anaesthesia. For patients with high preoperative residual urine, the mean catheterization time was 6.4 days (range: two to 28 days).

One patient developed disseminated intravascular coagulation due to previously undiagnosed prostate carcinoma, requiring a blood transfusion. After receiving four units, he was transferred to a private hospital and discharged five days postoperatively with the catheter removed and no further complications.

Another patient was admitted to the private hospital due to postoperative fever (temperature of 38.5°C). Intravenous antibiotics (gentamicin and ampicillin) were administered, resolving the fever within 24 hours. After 48 hours on gentamicin and ampicillin, he was discharged on amoxicillin/clavulanic acid for seven days. The patient was discharged home. No cases of "TURP syndrome" occurred, and there were no reported mortalities [3-5].

Eleven patients were re-catheterized because of recurrent retention secondary to neurogenic chronic retention. They were re-catheterized in the outpatient setting. The mean preoperative post-micturition residual volume in these patients was 651 mL. They were managed with continuous catheter drainage for four weeks and treated with bethanechol 40 mg four times a day (QID) orally starting 24 hours prior to catheter removal. After three weeks, they were weaned off the medication and continued to micturate satisfactorily.

Positive postoperative urine cultures were observed in 5.6% (63) of patients. There were no instances of sepsis or re-hospitalisations due to urine retention. The mean weight of resected tissue, as measured by the receiving pathology laboratory, was 22.48 grams (range: 7-81 grams). The average estimated blood loss was 67 ml (range: 20-600 ml) (Table 1). Additionally, one patient was admitted to a private hospital under the supervision of a cardiologist for monitoring of tachycardia, which spontaneously resolved, and no myocardial damage was diagnosed. 

**Table 1:** Patient statistics and duration of catheter insertion AUA: American Urological Association

Parameter	Mean (ranges)
Total patients	1,123
Age (years)	73.6 (49-91)
AUA score	22 (10-35)
Resection weight (grams)	22.48 (7-81)
Blood loss (millilitres)	67 (20-600)
Duration of catheter insertion	6.4 days (2-28)
Discharged same day (day surgery)	1,121 patients

## Discussion

The average length of stay in the hospital following a TURP in Australia can vary based on several factors, including the patient's overall health, the specific details of the procedure, and the hospital's protocols. In general, TURP is considered a minimally invasive procedure, and patients often experience a relatively short hospital stay compared to more extensive surgeries. Typically, patients undergoing TURP may stay in the hospital for one to three days, depending on their individual recovery progress and the postoperative care plan [3-6,8].

The traditional TURP procedure can be carried out as day surgery, allowing patients to be discharged without a catheter. The mean AUA symptom score of 22 highlights significant obstruction-related symptoms experienced by these patients before surgery [4]. In this series, there were three major complications requiring admission to an inpatient hospital (the development of disseminated intravascular coagulation (DIC), fever, and tachycardia). The administration of aspirin and clopidogrel was continued. Alternative anticoagulants were paused.

The key factor related to length of stay after TURP is the postoperative amount and duration of bleeding deemed acceptable for discharge [4,6-8]. Therefore, meticulous hemostasis is mandatory [7]. The use of catheter traction to assist with hemostasis is highly significant. It allows the basic principle of tamponade to be utilized to advantage.

There were no cases of “TURP syndrome,” which had previously implied the need for spinal anaesthesia [4]. However, as the spinal anaesthetic may take several hours to resolve, it was deemed more appropriate to use general anaesthesia. Thus, general anaesthesia has become the anaesthetic of choice. All patients had minimal postoperative pain, and it was unusual for them to require parenteral analgesia. 

Postoperatively, the patients were encouraged to drink fluids. An intravenous infusion of one litre of Hartmann’s solution was given during the procedure and a further one litre postoperatively. All patients were discharged home within 12 hours of hospitalization. Crucial to the success of same-day TURP is staff who are trained in the use of fluid balance charts, continuous catheter irrigation, and postoperative care in general. 

Patients were discharged under the supervision of a relative or friend who had participated in preoperative consultations and instruction sessions. For patients residing more than 100 km away or if it was deemed too late in the day for discharge, arrangements were made with a nearby resort facility (hotel).

The success of this procedure relied on careful planning, and establishing a consistent routine and care plan. Attention must be paid to preoperative preparation, perioperative hemostasis, and expert postoperative care. The shortened stay and early postoperative rehabilitation not only offer physical and psychological benefits to the patient but also result in potential economic advantages for both the patient and the community, contributing to an overall reduction in healthcare costs [8]. 

The cost implications for the hospital system between day surgery and a three-day admission in Australia are significant and multifaceted. Day surgery generally proves to be a more cost-effective option as it reduces the need for prolonged hospital stays, minimizing resources such as bed occupancy, nursing staff, and medical supplies [4,8]. This approach aligns with the broader trend in healthcare towards outpatient care and ambulatory procedures, aiming to optimize efficiency and reduce overall expenses. In contrast, a three-day admission involves sustained utilization of hospital resources, including extended bed occupancy, continuous monitoring by medical personnel, and a higher demand for ancillary services [8]. The financial impact extends beyond immediate expenses to include potential long-term effects, such as an increased risk of hospital-acquired infections during a longer stay. As healthcare systems strive for sustainability and efficiency, the balance between cost considerations and the delivery of quality patient care becomes a critical factor in shaping healthcare policies and practices.

## Conclusions

Performing catheter-free TURP on the same day has proven to be an effective and satisfactory approach for treating symptoms of lower urinary tract outflow obstruction. These procedures demonstrated minimal morbidity and no mortality. The shortened hospital stay contributed to a reduction in overall costs. Same-day TURP has emerged as a preferred method for addressing bladder outlet obstruction associated with BPH.

Day surgery for a TURP procedure offers several notable benefits compared to admission. Firstly, day surgery minimizes the length of hospital stay, reducing the demand for healthcare resources such as beds and staff. This approach aligns with the broader trend toward outpatient care, contributing to increased hospital efficiency and cost-effectiveness. Patients undergoing TURP in a day surgery setting often experience a quicker recovery in the familiar and less stressful environment of their homes, potentially lowering the risk of hospital-acquired infections. Additionally, the outpatient nature of day surgery may lead to decreased overall healthcare expenses for both the patient and the healthcare system. As healthcare systems continue to prioritize streamlined and patient-centred approaches, day surgery for TURP emerges as a viable and advantageous option.

## Figures and Tables

**Figure 1:**
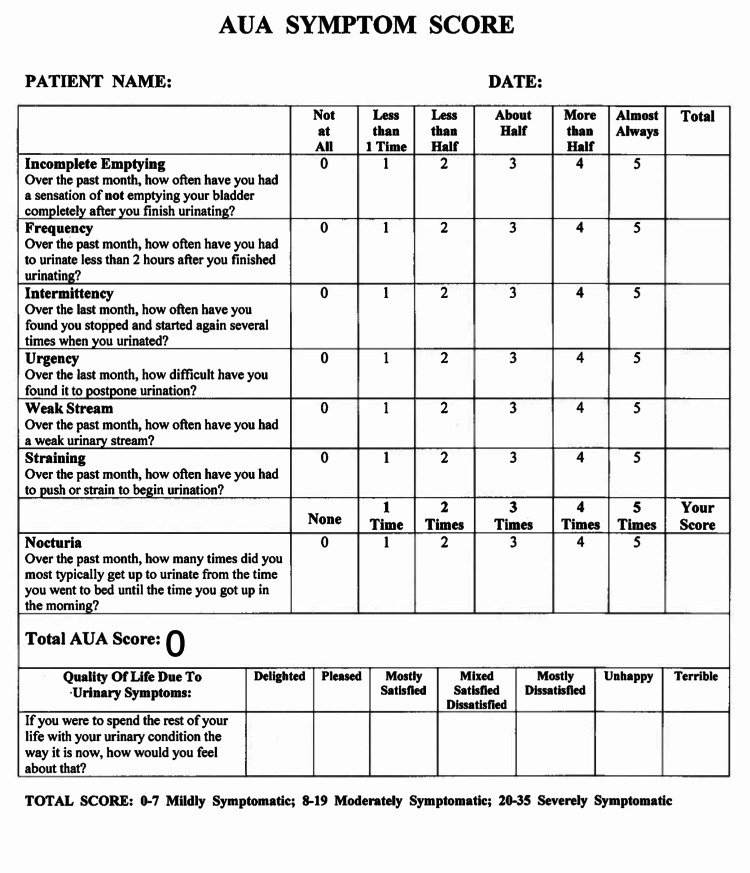
American Urological Association (AUA) scoring table Source: https://eirmc.com/util/documents/2019/eirmc-508_AUA_forms.pdf

## Appendices

Appendix one 
